# Recent Advances in the Removal of Organic Dyes from Aqueous Media with Conducting Polymers, Polyaniline and Polypyrrole, and Their Composites

**DOI:** 10.3390/polym14194243

**Published:** 2022-10-10

**Authors:** Jaroslav Stejskal

**Affiliations:** University Institute, Tomas Bata University in Zlin, 760 01 Zlin, Czech Republic; stejskal@utb.cz

**Keywords:** conducting polymers, composites, organic dyes, dye adsorption, dye removal, photocatalytic decomposition, polyaniline, polypyrrole

## Abstract

Water pollution by organic dyes, and its remediation, is an important environmental issue associated with ever-increasing scientific interest. Conducting polymers have recently come to the forefront as advanced agents for removing dye. The present review reports on the progress represented by the literature published in 2020–2022 on the application of conducting polymers and their composites in the removal of dyes from aqueous media. Two composites, incorporating the most important polymers, polyaniline, and polypyrrole, have been used as efficient dye adsorbents or photocatalysts of dye decomposition. The recent application trends are outlined, and future uses also exploiting the electrical and electrochemical properties of conducting polymers are offered.

## 1. Introduction

The removal of water-soluble organic dyes from aqueous media using composites based on conducting polymers has recently been reviewed [1]. The present article updates this research direction’s progress made in the last three years, 2020–2022. It is aimed at the organization of the recent studies by their objects of investigation rather than the compilation of experimental results. These are difficult to compare due to the widely differing experimental conditions, viz., the composition and concentration of adsorbents or photocatalysts, and the content of dye sorbates. Other reviews have recently been published on this topic and offer supplementary or alternative views [2,3,4].

**Scheme 1:**
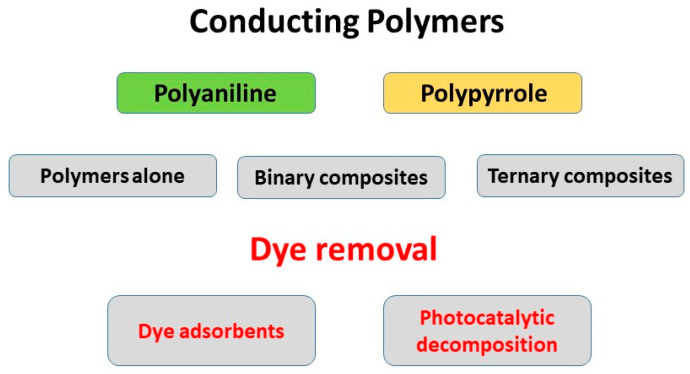
Conducting polymers, polyaniline, or polypyrrole, have recently been used alone or in the composites for organic dye removal from aqueous media by adsorption or photocatalytic decomposition.

Conducting polymers, such as polyaniline and polypyrrole (Scheme 1) and also, exceptionally, their substituted derivatives, have become an under consideration in the environmental sciences for water-pollution treatment. In principle, they can be used alone as powders. More often, however, they are applied as simple binary composites comprising an inorganic component, which provides some value-added, e.g., magnetic or photocatalytic properties. Alternatively, the composite component may be a natural or synthetic polymer affording the mechanical and material properties required by the applications, e.g., in the preparation of membranes or sponges. Finally, typical ternary composites include both inorganic and organic components, in addition to the conducting polymers.

The remediation of wastewater polluted by organic dyes is an important environmental goal. Conducting polymers are insoluble in aqueous media. When added to a solution of an organic dye, the reduction in the optical absorption reflecting the decreasing dye concentration has often been reported [1]. The process is classified as dye removal from the point of view of ecology (Scheme 1). Its mechanism is based on dye adsorption or its photocatalytic decomposition, or both occur simultaneously or in succession in various proportions.

**Figure 1:**
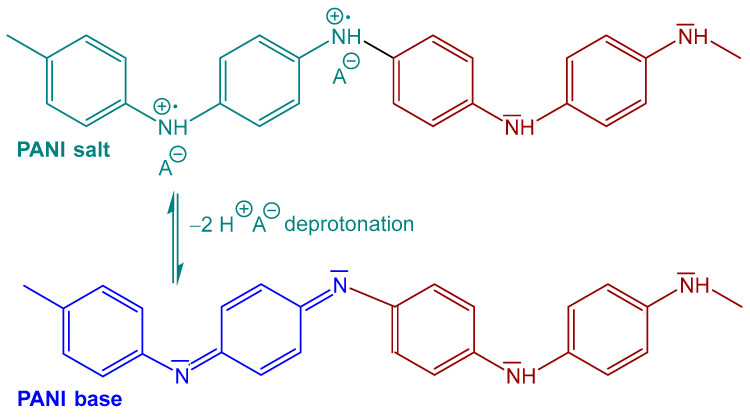
Polyaniline salt and base. A^−^ is a counter-ion, usually chloride or sulfate.

Polyaniline [5] (Figure 1), probably the most common conducting polymer, is typically prepared by the oxidation of aniline in an acidic aqueous medium with ammonium peroxydisulfate [6]. The polymerization starts with common chemicals and proceeds easily at room temperature, in the open air, within tens of minutes, and at a stoichiometric yield. The conducting polyaniline salt (Figure 1) has an electronic conductivity in the units of S cm^−1^. The ease of preparation at an economic cost makes polyaniline an attractive object of application. Its hydrophilic, thin films have an emerald-green color. Under alkaline conditions, polyaniline salt converts to a nearly hydrophobic non-conducting blue base; the transition occurs at pH 4–6. For that reason, the interaction with organic dyes will be pH-dependent. On the other hand, most of the studies refer to the neutral conditions met in practice.

**Figure 2:**
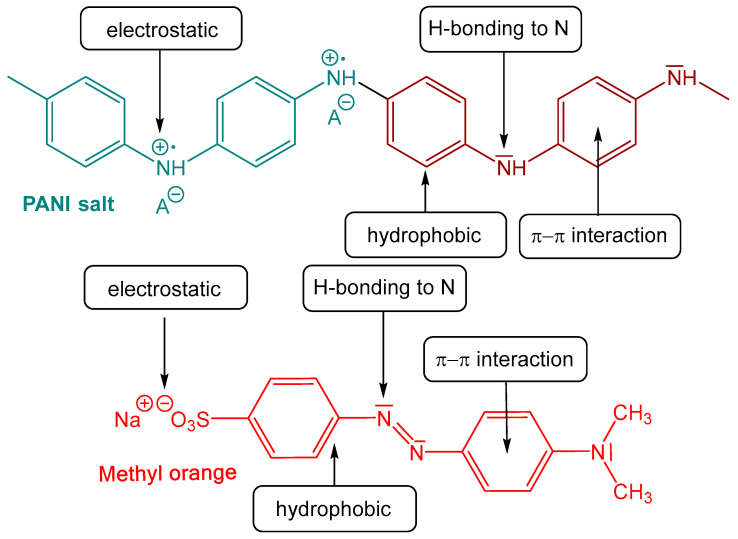
A conducting polymer, polyaniline, and an organic dye, methyl orange, and sites of their possible interactions.

Conducting polymers, and many organic dyes, illustrated on polyaniline and methyl orange (Figure 2), share a similar molecular structure. They have a system of conjugated single and double bonds that are responsible for their color. They typically contain aromatic benzenoid or quinonoid rings. Nitrogen atoms, in their structure, are able to be hydrogen bonded. In addition to the hydrophobic organic section, the dyes include a charged ionic group that affords solubility in water. The same applies to the conducting polymers, which, however, are insoluble in water due to extensive intermolecular hydrogen bonding.

**Figure 3:**
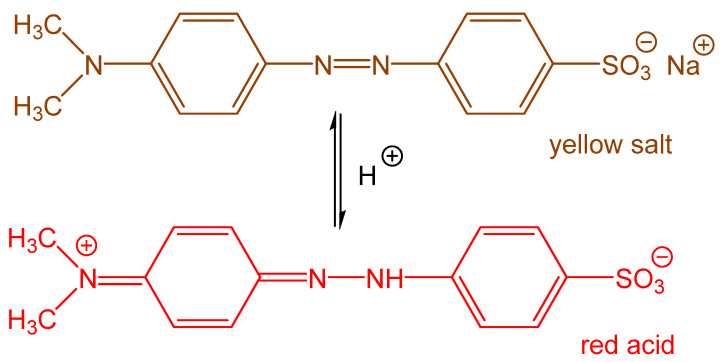
Yellow methyl orange salt converts to red acid under acidic conditions.

Many organic dyes are used as acidobasic indicators. For example, the yellow color of methyl orange changes under neutral pH conditions to red when acid is added, with the transition range being pH 3.1–4.4 (Figure 3). However, not only does the color change but the molecular structure and hydrophobicity are also altered simultaneously; while the salt is well soluble, the acid form is not. This means that the interactions between polyaniline and methyl orange will occur between different species under different pH. This fact should be kept in mind. Methyl orange is also one of the common azo dyes extensively used in textile, paper, printing, and food industries and often contaminates industrial wastewater.

The interactions between the organic dyes and conducting polymers are responsible for the dye removal from aqueous media [1,7]. The *adsorption* of dyes on conducting polymers has often been reasonably proposed as the most relevant possibility. Adsorption is a surface phenomenon that occurs when the dye is expected to be attached to the conducting polymer surface by physical van der Waals forces or due to physicochemical interactions, e.g., of the π–π electron type. Conducting polymers have a typical specific area of tens m^2^g^−1^ [8], far lower than classical adsorbents when this parameter is one or two orders of magnitude higher. The adsorption, thus, probably cannot be the exclusive mechanism of efficient dye removal. The dye *absorption* is thus likely to occur after the dye molecules diffuse into the conducting polymer phase, a process much slower compared with the adsorption. Conducting forms of polyaniline and polypyrrole are hydrophilic and penetrable with an aqueous phase.

There is a choice of attractive interactions that occur, both during the adsorption or absorption. One possibility is the *π–π interaction* between the aromatic rings present in typical dyes and conducting polymers. The *electrostatic interactions*, when a soluble anionic dye produces insoluble salt with the conducting polymers, also need to be considered. The *hydrogen bonding* of hydrogen atoms in conducting polymers to nitrogen atoms in dyes or vice versa is probably one of the strongest interactions to account for. Finally, the *hydrophobic interactions*, when the non-ionic parts of conducting polymers (Figure 1) and dyes prefer to contact each other instead of with water, are analogous to the formation of surfactant micelles. Indeed, water-soluble dye molecules composed of a hydrophobic body and ionic group conform to the definition of surfactants.

The *photocatalytic decomposition* of dyes has also often been reported in the literature. In this case, the dye is removed by the degradation process to colorless products with the help of photogenerated active peroxide species. Such a mechanism is relevant when the dye removal occurring in the dark and when exposed to illumination considerably differ, the latter being faster [9]. Synergistic dye adsorption is usually also operational in these experiments [10]. The fact that the conducting polymers are colored, i.e., they efficiently absorb the visible light, which may hinder the light penetration to the photocatalyst interior, has not been discussed.

**Figure 4:**
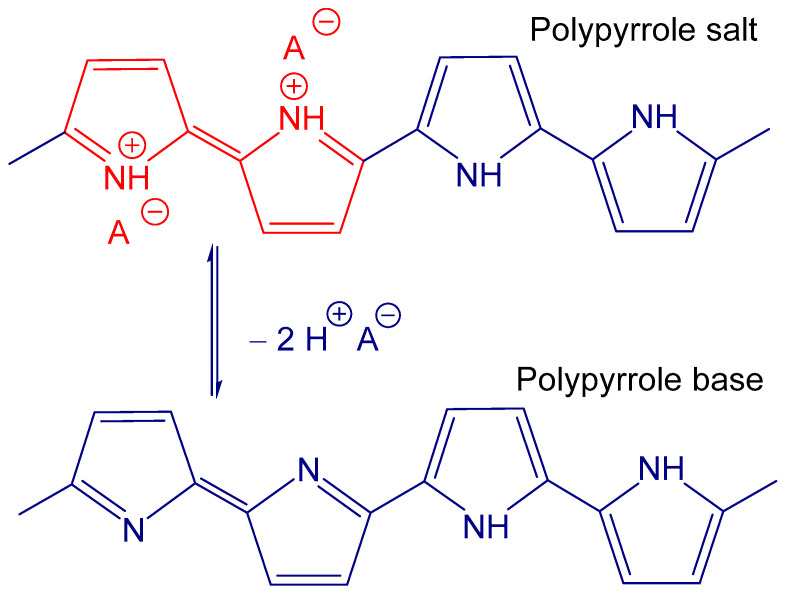
Polypyrrole salt and base. A^−^ is a counter-ion, usually chloride or sulfate.

Polypyrrole is another conducting polymer (Figure 4). Its common globular form has a conductivity comparable to polyaniline, the units of S cm^−1^. The transition between the conducting salt and the less conducting base is shifted to a much lower pH [11]. The adsorption is thus likely to depend on the pH [12]. Under neutral pHs, most often met in practice, polypyrrole maintains its conducting form, whereas polyaniline may start to lose its conductivity due to deprotonation (Figure 1).

**Figure 5:**
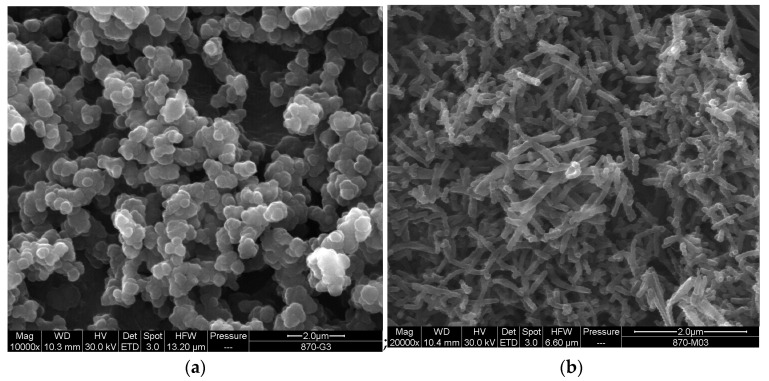
Globular polypyrrole (**a**) and polypyrrole nanotubes (**b**).

Polyaniline and polypyrrole have a typical globular morphology (Figure 5). In contrast to polyaniline, polypyrrole converts its morphology from a globular to a nanotubular state (Figure 5) when its preparation occurs in the presence of methyl orange dye. At the same time, the conductivity of polypyrrole increases by one order of magnitude to tens S cm^−1^ [13]. The morphology and conductivity of polypyrrole may be controlled by introducing various dyes, again in contrast to polyaniline [14]. Especially in the case of polypyrrole, it is important to distinguish between the globular and nanotubular forms that behave differently with respect to the dye removal [15,16].

**Figure 6:**
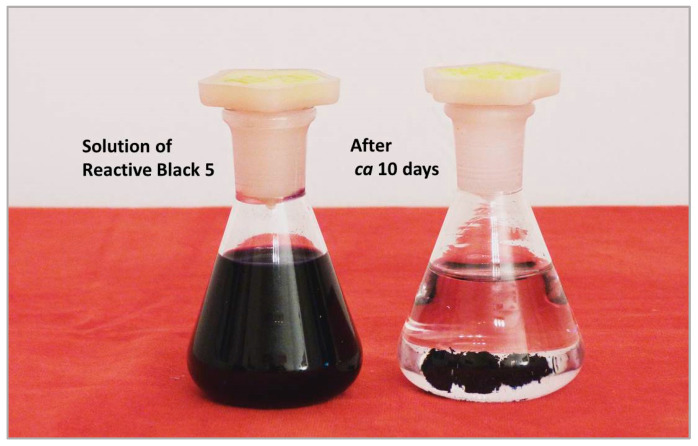
The solution of Reactive Black 5 before (**left**) and after dye adsorption by polypyrrole nanofibers (**right**). Adapted from [16].

**Figure 7:**
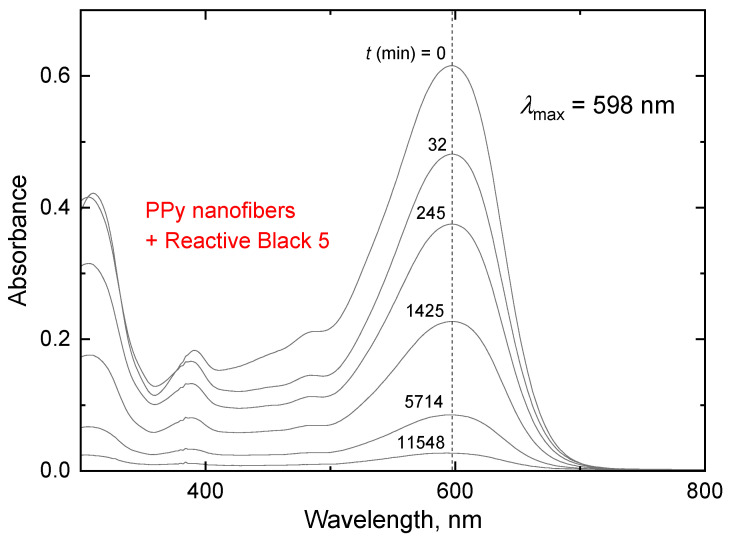
The decrease in the optical absorbance of the Reactive Black 5 solution with time, *t*. An amount of 5 mg of Reactive Black 5 and 50 mg of polypyrrole nanofibers in 50 mL of water at 20 °C [16].

**Figure 8:**
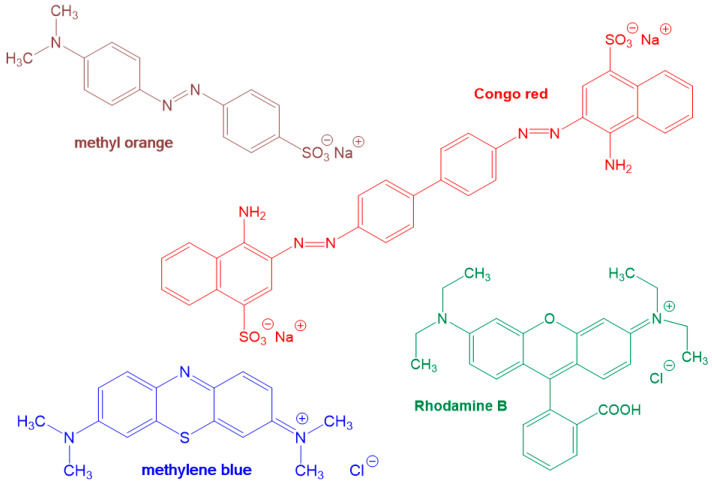
The most often used anionic (**top**: methyl orange, Congo red) and cationic dyes (**bottom**: methylene blue and Rhodamine B).

Another reason for the numerous studies reporting dye removal (Figure 6) [17,18] is the ease of the experimental procedure based on the UV-visible spectroscopy that monitors the optical absorption of dye solution in time after the addition of a conducting polymer or its composite (Figure 7). Such removal experiments have been carried out under static conditions and only exceptionally in a dynamic mode [19]. The most often used dyes used in the experiments are methyl orange, Congo red, methylene blue, and Rhodamine B (Figure 8).

The experimental data and the time dependence of the dye concentration decrease are often interpreted in terms of various adsorption models: a pseudo-first-order, a pseudo-second-order, Freundlich, Langmuir, Temkin, or others. The authors found that one of them provided the best fit of the data, with the Langmuir isotherm being the most often winner [7,8,20,21,22,23,24]. This is the correct approach, but only if the dye removal mechanism is based exclusively on adsorption.

Only the removal of soluble organic dyes is reviewed below; however, the many adsorbents and experimental techniques can also be used for the removal of colored toxic heavy metal compounds [3], such as lead(II) and cadmium(II) salts [25], chromium(VI) [26,27,28,29], and zinc(II) ions [30]. This also applies to the removal of various drugs, which may often be structurally related to the dyes, e.g., oxytetracycline [31], chloramphenicol or furazolidone [32], rifampin [33] as well as organic dyes. Such studies illustrate a more general application of the composites based on conducting polymers in waste water remediation.

## 2. Polyaniline Adsorbents

Polyaniline alone has been recently tested as an adsorbent in several papers. It was applied to the adsorption of cationic dye, Basic Red 46 [20]. Polyaniline, prepared with two different oxidants, ammonium peroxydisulfate, and manganese dioxide, hesitantly adsorbed methylene blue to the same extent and more efficiently than Reactive Black 5 [34]. Polyaniline, prepared with iron(III) chloride oxidant, was used to adsorb Acid Red G [35]. Another type of polyaniline, prepared in the absence of acid and containing aniline oligomers, adsorbed methylene blue and indigo carmine [36]. Polyaniline, prepared in the presence of amino acids, was tested for the adsorption of Congo red [37]. The same dye and Rhodamine B were adsorbed with hollow polyaniline microspheres [38]. A polyaniline base was used for the adsorption of methylene blue [39]. The reports on the adsorption of neat polyaniline as a reference material can also be found in various papers dealing with polyaniline composites, e.g., [9,22,40,41].

### 2.1. Binary Polyaniline Composites

Conducting polymers are useful for the fundamental investigation of interactions with conducting polymers. For practical applications, however, conducting composites are required. This means that additional material is introduced to the conducting polymers that afford mechanical or other material properties suitable for the particular application, such as gel-like or macroporous substances [16,42,43]. They affect the distribution of conducting polymer within the composite and the access of organic dyes to adsorption sites. The independent adsorption of dyes on the supporting substrate must also be considered. The non-conducting component may act as an independent adsorbent, e.g., melamine sponge [16], that may improve or broaden dye removal ability.

The simple binary composites are usually prepared by an in situ deposition of conducting polymers on the substrates immersed in a reaction mixture used for conducting polymer preparations, i.e., they are of a core–shell type (Table 1). This is based on the observation that any material in contact with the reaction mixture becomes coated with a thin submicrometre film [44], which is well suited for adsorption purposes. The coating of inorganic substrates is represented by the following: barium titanate [45]; cobalt ferrite [28]; cobalt sulfide [46]; copper(II) oxide [47]; fly ash [48]; iron oxyhydroxide [49]; iron sulfide [21]; magnetite [50]; manganese(IV) dioxide [34,40]; manganese ferrite [51]; magnesium ferrite [52]; molybdenum trioxide [53]; montmorillonite [54]; nickel oxide [40]; silica gel [55]; tin dioxide [56]; titanium dioxide [22,33,57,58]; zeolite [59]; zinc oxide [60,61,62]; zirconium(IV) dioxide [63].

A separate class of adsorbents is represented by the polyaniline-coated carbonaceous materials, e.g., activated carbon [7,64]; activated carbonized peanut shells [65]; activated carbons based on prickly pear seeds [66]; carbonized tea waste [67]; graphene [47,68]; multiwall carbon nanotubes [8] or reduced graphene oxide [30,69,70]. Only exceptionally, polyaniline was decorated in a reverse manner by an inorganic component, such as copper(II) oxide [71].

It should be mentioned that some inorganic compounds, typically titanium dioxide or zinc oxide but also others, are also active as photocatalysts. This means that the dye removal reported as adsorption may contain a contribution of photocatalytic decomposition. This also applies vice versa: prior adsorption of the dye on a photocatalyst is needed for photocatalytic degradation. For the practical application, it need not be important which mechanism of dye removal, adsorption, or photodegradation predominates.

The organic substrates coated with conducting polymers include synthetic polymers, such as carboxymethylcellulose gel [78], Kevlar fibers [73], macroporous melamine sponges [16], poly(ethylene oxide) [76], polyimide membrane [74], polystyrene [64], poly(vinyl alcohol) aerogel [42], polyurethane foam [77], and various natural materials, such as [24,75], chitosan [82], opuntia ficus [80], almond and wall nut shells [81] or tea saponin [41]. Only exceptionally, both components were simply mixed, e.g., polyaniline and nitrogen-containing carbon nanodots [87] or zinc oxide [61]. In this case, the fact that the organic component may also be an efficient adsorbent in addition to conducting polymer also has to be kept in mind [16].

**Figure 9:**
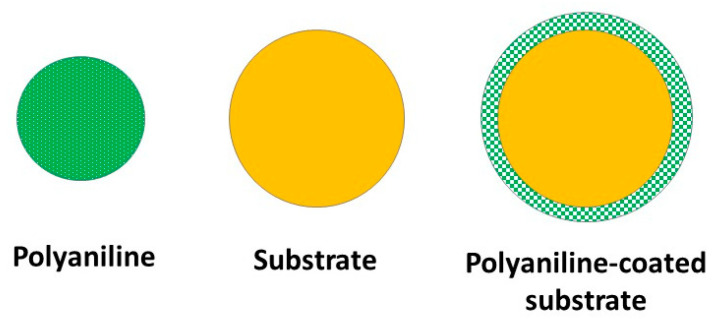
Globular polyaniline and polyaniline-coated substrate.

The removal of dyes by the hybrid inorganic–organic composites was more efficient when compared with neat polyaniline and inorganic component alone [7,8,41,72,88]. This is interpreted as a synergistic effect, but a few words of explanation are relevant. Polyaniline is prepared in globular form (Figure 9) with closely packed chains cross-linked by hydrogen bonding to nitrogen atoms. When polyaniline is deposited on a particulate substrate in situ during the polymer preparation, thin submicrometre polyaniline film grows on the substrate’s surface. It is assumed that the polyaniline chains are more organized and grow in a perpendicular direction to the coated surface. The resulting brush-like morphology may be better penetrable to the dye molecules, and also, the specific surface area of polyaniline in coatings will be larger than that in the compact globules. For this reason, the hybrid coatings perform better as adsorbents than individual components. The observed “synergism” is not a result of a specific interaction between the polyaniline and supporting materials, but it is due to the different morphology and random chain arrangement in the globular polyaniline and more ordered structure in the polyaniline coatings.

### 2.2. Ternary Polyaniline Composites

Multicomponent composites are composed of more than two components. Here, three is a typical number (Table 1). For example, they include some specific parts, such as magnetite or ferrites [26,42,46,48,50,51,53,82,89,90], which serve the adsorbent separation with a magnetic field.

Poly(vinyl alcohol) supports the composite macroporosity when used in aerogels [42,43]. Calcium alginate hydrogel provided a matrix for polyaniline/sawdust [86]. A supporting polymer was also introduced when the adsorbent was a part of a membrane [40,74,76,79,83,84,85].

Many studies agree that polyaniline is an efficient adsorbent or photocatalyst for both anionic and cationic dyes [24,55,91,92], meaning that ionic interactions are not decisive in their activity.

## 3. Polyaniline Photocatalysts

There is a single report on the photocatalytic degradation of malachite green and methylene blue caused by polyaniline [93]. Polyaniline, however, was prepared by the oxidation of aniline with periodic acid in acetonitrile and not by the standard way using ammonium peroxydisulfate in an aqueous medium [5].

### 3.1. Binary Polyaniline Composites

The composites used for the photocatalytic decomposition of dyes have also displayed adsorption properties [94] because dye adsorption on a photocatalyst is the prerequisite for efficient photocatalytic dye decomposition. The composites typically contained an inorganic photocatalyst, a conducting polymer, and a supporting material (Table 2). The inorganic component was coated with conducting polymer in situ during the synthesis of polyaniline (Figure 9). A polyaniline coating has a typical thickness of 100–200 nm and is green; however, it is still sufficiently transparent to all wavelengths of the UV-visible light, which thus has access to the photocatalyst. This is important for the success of photocatalysis afforded by hybrid composites.

The list of simple polyaniline composites includes the following moieties: bismuth oxychloride [95]; cadmium sulfide [96,97]; carbon nitride [98]; magnetite [89]; multiwall carbon nanotubes [99]; nickel [100]; reduced graphene oxide [101]; silver molybdate [29]; silver oxide [102]; tin dioxide [103]; titanium dioxide [104,105,106]; tungsten trioxide [107]; zinc oxide [61,104,108,109,110]; zirconium dioxide [9].

In the preparation of the above composites, the inorganic substrate was coated with polyaniline in the course of its preparation. On rare occasions, polyaniline was simply mixed with an inorganic compound. The mixture of polyaniline and nitrogen-containing carbon nanodots, obtained by the hydrothermal method from bovine serum albumin, photocatalyzed the decomposition of various dyes [87]. A mixture of commercial polyaniline with nickel tungstate was used to decompose methylene blue and crystal violet [111].

### 3.2. Ternary Polyaniline Composites

Ternary systems are more useful for practical applications (Table 2) but also more difficult to interpret because each component can participate in dye adsorption. The latest examples are: attapulgite/*g*-carbon nitride [112]; bismuth(III) oxyiodide/reduced graphene oxide [10]; cerium dioxide/polystyrene [113]; cobalt/zinc ferrite [90]; sodium bismuthate/polycarbonate [114]; titanium dioxide/cadmium sulfide [97]; titanium dioxide/chitosan [115]; titanium dioxide/poly(vinylidene fluoride) [84,85]; zinc oxide/graphene oxide/viscose [116]; zinc/magnetite [117,118].

The antibacterial activity of the polyaniline composites has occasionally been mentioned [56,75,78,101]. This may be expected, especially in photocatalytic experiments, due to the formation of reactive oxygen species [87,119,120].

## 4. Polypyrrole Adsorbents

The adsorption or an anionic dye, Reactive Black 5, on globular polypyrrole was poor, but it was complete when the polypyrrole nanofibers of nanotubes were used instead [16]. In another study, the adsorption of both anionic and cationic dyes was attributed to electrostatic interactions and hydrogen bonding [12].

### 4.1. Binary Polypyrrole Composites

In contrast to polyaniline, polypyrrole composites have been investigated as adsorbents less often (Table 3). Polypyrrole has been combined and, as a rule, deposited in situ during the polymerization of pyrrole onto inorganic substrates, such as carbon nitride [121], MXene [122], nanosilica [23], and stainless steel mesh [123].

Organic supports, such as melamine sponge [16] and poly(*p*-phenylene terephthalamide) [124], have been used to provide macroporous sponges or membranes. Additionally, natural organics have been used to provide a value-added material, e.g., bacterial cellulose [125], carbonized chicken feathers [126], Chinese yam peel [127], and wheat straw [128].

In the systems comprising polypyrrole, the morphology of this polymer, as globules or nanotubes (Figure 5), has to be taken into account. Globular polypyrrole, deposited on macroporous melamine sponge, did not significantly adsorb Reactive Black 5 [16], but polypyrrole nanotubes on the same substrate proved to be an excellent adsorbent of this dye [15,16]. This may be the result of a better polymer chain organization in nanotubes compared to the random arrangement in globules, similar to polyaniline (Figure 9). The polypyrrole nanotubes had a higher conductivity, specific surface area, and stability with respect to the acidity changes when compared with the globular form [11,13]. The adsorption decreased after coating with polypyrrole. Melamine sponge alone was not indifferent; however, it efficiently adsorbed a cationic dye, crystal violet, meaning that the adsorption properties of the support also have to be considered.

In general, only limited attention has been paid to the composites of polypyrrole nanotubes. Wheat straw biomass was coated in situ with the polypyrrole nanotubes and used for the removal of Eosin Y [128]. The polypyrrole nanotubes were dispersed in sodium alginate gel and then applied for the removal of methylene blue [129].

### 4.2. Ternary Polypyrrole Composites

The typical adsorbents had three components again: a conducting polymer, an inorganic compound, and a supporting material (Table 3). Cobalt oxide/graphene [25] and molybdenum trioxide/magnetite [53] are examples of inorganic components, while the organics are represented by sodium alginate/algae biomass [130], polyacrylonitrile/poly(*N*-vinylpyrrolidone) [131], and poly(ethyleneglycol methacrylate)/magnetite microspheres [132].

## 5. Polypyrrole Photocatalysts

The photocatalytic activity of polypyrrole alone was reported only rarely and was demonstrated in the removal of methyl orange [133] and malachite green [134]. The main reactive species generated under illumination were identified to be oxygen anion-radicals.

### 5.1. Binary Polypyrrole Composites

In addition to polypyrrole, the simple binary composites contained inorganic components (Table 4). The improvement in the catalytic activity has often been observed after the surface modification with a conducting polymer.

The substrates used for the deposition of polypyrrole were as follows: iron [134]; iron tungstate [135]; molybdenum sulfide [92]; MWCNT [136]; silver [137]; silver manganite [32]; titanium dioxide [58]; or tungsten trioxide [138].

### 5.2. Ternary Polypyrrole Composites

Most studies, however, have been devoted to multicomponent composite materials (Table 4). The ternary composites typically included photocatalytically active inorganic compounds, e.g., titanium dioxide or zinc oxide. A third component has often been included, typically a polymer, to provide materials with a controlled morphology or mechanical properties required by applications.

A list of the composites used in the recent literature includes: graphitic carbon/reduced graphene oxide composites [119]; multiwall carbon nanotubes/polyacrylonitrile [139]; silver/tin oxide [120]; titanium dioxide/Cu-MOF [91]; titanium dioxide/graphene oxide [140]; zinc oxide/activated carbon [72]; zinc oxide/cellulose acetate [141,142]; zinc oxide/copper [143]; or zinc oxide/magnetite [19].

## 6. Polyaniline and Polypyrrole Derivatives

Substituted polyanilines have been used in dye removal, but only exceptionally. The poly(*N*-methylaniline)/chitosan composite was used for methyl red removal [144]. Polyphenylenediamines [145] are promising candidates for adsorbents due to their preparation from non-toxic monomers at an economical cost. They are considerably less conducting compared with polyaniline, but this need not be a drawback in dye removal. Poly-*p*-phenylenediamine, deposited on hydrolyzed polyacrylonitrile membrane, has recently been reported to remove the anionic Congo red and Direct Red 23 dyes [146].

Polyaniline can easily be converted to nitrogen-containing carbons [147]. The morphology is retained after this process, except for some shrinkage. Carbon fibers prepared from the polyaniline precursor were combined with titanium dioxide to produce a photocatalyst for the decomposition of methylene blue [148].

The study that combined both of our reviewed conducting polymers, polyaniline, and polypyrrole, in the composite with carbon nitride involved methylene blue as the decomposition object [149].

Concerning polypyrrole, there are many ways to modify the polymer chains by copolymerization with ring-substituted monomers; however, reports in this direction are scarce. For example, poly(pyrrole-*co*-sulfophenylenediamine) was found to adsorb an anionic dye, Congo red, and a cationic dye, methylene blue [150].

Polypyrrole was exposed to 200, 400, and 650 °C in an inert atmosphere. This first deprotonated polypyrrole (Figure 4), followed by its carbonization [151,152]. The ability to remove Reactive Black 5 from aqueous media decreased with an increasing exposure temperature. The activation of polypyrrole with potassium hydroxide during pyrolysis only slightly increased the adsorption ability [152].

## 7. Conclusions

The conducting polymers, polyaniline, and polypyrrole, became well-established active materials for wastewater management. In recent years, from 2020 to 2022, most papers have been dedicated to the application of polyaniline as a dye adsorbent (Table 1). A preference was given to the anionic dyes, where the electrostatic interaction with polymer cations was expected. In the photocatalytic studies, the participation of the anionic and cationic dyes was balanced (Table 2). The number of papers reporting on polypyrrole was slightly lower with regards to the comparable treatment of anionic and cationic dyes both in adsorption (Table 3) and photocatalytic decomposition (Table 4). Still, more detailed studies on neat polyaniline and polypyrrole, even though of academic interest, would be welcome to further our understanding of the role of these polymers in the composites for practical application. Concerning the mechanism of dye removal, dye adsorption, or photocatalytic decomposition, they are likely to be operative at the same time, and their contributions should be estimated if possible. It has to be stressed that the present review concerns the papers published in 2020–2022. For the extensive work reported in preceding years, the readers are referred to an earlier review [1].

## 8. Future Prospects

Conducting polymers are not just conducting [153]. Their use as adsorbents of organic dyes is a typical example of an application that does not require electrical conductivity. However, in the next step, their conductivity allows for the construction of electrodes by their in situ deposition on non-conducting substrates and subsequent use in electrochemistry. In this experiment, an electric potential was applied to the conducting polymer adsorbent. The electrochemical switching between the redox polymer forms may change the adsorption properties due to a change in molecular structure, degree of protonation, and/or hydrophilicity. For example, the electroremediation of aqueous effluents containing Congo red on polyaniline/titanium dioxide composite points in this direction [58], similar to the electrooxidation of this dye on the polyimide membrane coated with polyaniline [74]. Such systems might be regarded as intelligent adsorbents controlled by the applied potential.

The array of adsorbents may be broadened by testing the polymers of ring-substituted anilines, viz., phenylenediamines [145] and various copolymers, with aniline. As a rule, they have a lower conductivity than polyaniline, but this would not limit their suitability for dye removal experiments. Concerning polypyrrole, attention should be paid to its morphology, especially to the nanotubes, which have been tested only marginally.

## Figures and Tables

**Table 1:** Polyaniline composites applied as dye adsorbents ^a^.

Dye		PANI Composite with	Reference
BINARY COMPOSITES			
Acid Blue 74	−	Tea saponin	[41]
Acid Red 18	−	CoFe_2_O_4_	[28]
Brilliant green	+	Reduced graphene oxide	[69]
Brilliant green	+	SiO_2_	[55]
Congo red	−	Activated carbon	[66]
		BaTiO_3_	[72]
		Graphene	[68]
		Kevlar fibers	[73]
		Polyimide	[74]
		SiO_2_	[55]
		Tea saponin	[41]
		TiO_2_	[58]
		ZnO	[60]
Crystal violet	+	SiO_2_	[55]
Direct Blue 14	−	Activated carbon	[64]
		Polystyrene	[64]
Direct Blue 15	−	SnO_2_	[56]
Eosin Y	−	FeS	[21]
Methyl orange	−	Activated carbon	[7]
		Bacterial cellulose	[75]
		Cellulose	[24]
		CuO	[71]
		Fe_3_O_4_	[50]
		Manganese ferrite	[51]
		Multiwall carbon nanotubes	[8]
		PEO	[76]
		Polyurethane foam	[77]
		TiO_2_	[22]
Methyl red	−	MgFe_2_O_4_	[52]
Methylene blue	+	Boron cluster	[27]
		Carbonized peanut shells	[65]
		CMC	[78]
		MnO_2_	[34]
		Poly(phenyl sulfone)	[79]
		Reduced graphene oxide	[30]
		ZnO	[60]
		ZnO/SiO_2_	[62]
		ZrO_2_	[63]
		Cellulose	[24]
Mordant Blue 9	−	CMC	[78]
Orange G	−	Opuntia ficus	[80]
		Nut shells	[81]
		Zeolite	[59]
Orange II	−	FeO(OH)	[49]
Reactive Black 5	−	Manganese dioxide	[34]
Reactive Green 19	−	Montmorillonite	[54]
4Rhodamine 6G	+	Boron cluster	[27]
Rhodamine B	+	Carbonized tea waste	[67]
		CMC	[78]
Sunset yellow	−	Reduced graphene oxide	[30]
			
TERNARY COMPOSITES			
Allura Red	−	Bentonite/PEO	[40]
Congo red	−	CoS/graphite	[46]
		Fly ash/Fe_3_O_4_	[48]
		L-cysteine/reduced graphene oxide	[70]
Crystal violet	+	Fe_3_O_4_/chitosan	[82]
Methyl orange	−	Cu/TiO_2_	[22]
		Fe_3_O_4_/chitosan	[82]
		Fly ash/Fe_3_O_4_	[48]
		MnO_2_/NiO	[40]
		Montmorillonite/PVAL	[43]
		PAA/PAN	[83]
		SiO_2_	[55]
		TiO_2_/PVDF	[84]
		TiO_2_/PVDF	[85]
Methylene blue	+	MoO_3_/Fe_3_O_4_	[53]
		PAA/PAN	[83]
Orange G	−	Calcium alginate/sawdust	[86]
Reactive Black 5	−	Montmorillonite/PVAL	[43]
		Fe_3_O_4_/PVAL	[42]
Reactive Orange 5	−	TiO_2_/zeolite	[33]
Rhodamine B	+	CuO/graphene	[47]
Safranin	+	Montmorillonite/PVAL	[43]

^a^ The plus sign + denotes a cationic dye, and the minus sign − is an anionic one. The same notation is used in all Tables. CMC = carboxymethylcellulose; PAA = poly(acrylic acid); PAN = polyacrylonitrile; PEO = poly(ethylene oxide); PVAL = poly(vinyl alcohol); PVDF = poly(vinylidene fluoride).

**Table 2:** Polyaniline composites applied as photocatalysts.

Dye		PANI Composite with	Reference
BINARY COMPOSITES			
Acid Blue 29	−	CdS	[97]
Acid Orange 52	−	TiO_2_	[105]
Brilliant green	+	Ni	[100]
Congo red	−	Carbon nanodots	[87]
		*g*-C_3_N_4_	[98]
		Reduced graphene oxide	[101]
		ZrO_2_	[9]
Crystal violet	+	Carbon nanodots	[87]
		NiWO_4_	[111]
Disperse Red 1	−	Co_0.5_Zn_0.5_Fe_2_O_4_	[90]
Fluorescein	−	ZnO	[108]
Levafix red	−	Fe_3_O_4_	[89]
Malachite green	+	Fe_3_O_4_	[89]
Methyl orange	−	Ag_2_O	[102]
		*g*-C_3_N_4_	[98]
		MWCNT	[99]
		TiO_2_	[106]
Methylene blue	+	Carbon nanodots	[87]
		MWCNT	[99]
		Nb_2_O_5_	[101]
		NiWO_4_	[111]
		TiO_2_	[104]
		ZnO	[61]
Orange II	−	ZnO	[109]
Reactive Blue 220	−	BiOCl	[95]
Reactive Orange 14	−	Co_0.5_Zn_0.5_Fe_2_O_4_	[90]
Rhodamine B	+	Carbon nanodots	[87]
		CdS	[96]
		MWCNT	[99]
		SnO_2_	[103]
		WO_3_	[107]
			
TERNARY COMPOSITES			
Acid Blue 29	−	TiO_2_/CdS	[97]
Allura red	−	TiO_2_/PVDF	[84]
Congo red	−	Zn/Fe_3_O_4_	[118]
Methyl orange	−	Ag/AgMoO_4_	[29]
		*g*-C_3_N_4_/attapulgite	[112]
		TiO_2_/chitosan	[115]
		TiO_2_/PVDF	[84]
Methyl red	−	ZnO/Cu/Ni	[110]
Methylene blue	+	CeO_2_/polystyrene	[113]
		TiO_2_/chitosan	[115]
		ZnO/graphene oxide	[116]
Methylene green	+	NaBiO_2_/polycarbonate	[114]
Reactive Black 5	−	TiO_2_/Fe_3_O_4_	[117]
Reactive Brilliant Red K-2K	−	Graphene oxide/cellulose	[94]
Rhodamine B	+	BiOI/reduced graphene oxide	[10]
		NaBiO_2_/polycarbonate	[114]

**Table 3:** Polypyrrole composites applied as dye adsorbents ^a^.

Dye		Composite with	Reference
BINARY COMPOSITES			
Acid Orange 7	−	nanosilica	[23]
alizarine red	−	carbonized chicken feathers	[126]
chrysoidine	+	melamine	[16]
Congo red	−	Chinese yam peel	[127]
		PPT	[124]
Eosin Y	−	PPT	[124]
		wheat straw	[128]
fuchsin	−	bacterial cellulose	[125]
malachite green	+	steel mesh	[123]
methyl orange	−	MXene	[122]
methylene blue	+	*g*-C_3_N4	[121]
		MXene	[122]
		sodium alginate	[129]
Reactive Black 5	−	melamine	[15,16,17,18]
Rhodamine B	+	*g*-C_3_N4	[121]
		steel mesh	[123]
			
TERNARY COMPOSITES			
Acid Orange 7	−	PAN/PVP nanofibers	[131]
Acid Yellow 9	−	PAN/PVP nanofibers	[131]
Congo red	−	CoO/graphene	[25]
		PEGMA/Fe_3_O_4_	[132]
		sodium alginate/algae biomass	[130]
metanil yellow	−	PAN/PVP nanofibers	[131]
methyl orange	−	magnetite/chitosan	[26]
methylene blue	+	CoO/graphene	[25]
		MoO_3_/Fe_3_O_4_	[53]

^a^ PAN = polyacrylonitrile; PVP = poly(*N*-vinylpyrrolidone); PEGMA = poly(ethyleneglycol methacrylate); PPT = poly(*p*-phenylene terephthamide).

**Table 4:** Polypyrrole composites applied as dye photocatalysts.

Dye		Composite with	Reference
BINARY COMPOSITES			
alizarine red	−	FeWO_4_	[135]
Congo red	−	MoSe_2_	[92]
malachite green	+	Fe	[134]
methyl orange	−	AgMnO_2_	[32]
		TiO_2_	[58]
methylene blue	+	Ag	[137]
	+	MWCNT	[136]
	+	WO_3_	[138]
Rhodamine B	+	MoSe_2_	[92]
	+	MWCNT	[136]
rose bengal	−	FeWO_4_	[135]
			
TERNARY COMPOSITES			
brilliant red	−	*g*-C_3_N_4_/reduced graphene oxide	[119]
Congo red	−	Ag/Sn_3_O_4_	[120]
malachite green	+	Ag/ZnO	[142]
	+	Ag/ZnO/cellulose acetate	[141]
metanil yellow	−	CuO/ZnO	[143]
methyl orange	−	MWCNT/polyacrylonitrile	[139]
		TiO_2_/Cu-MOF	[91]
methylene blue	+	TiO_2_/graphene oxide	[140]
		Ag/Sn_3_O_4_	[120]
		TiO_2_/Cu-MOF	[91]
		ZnO/activated carbon	[141]
		ZnO/Fe_3_O_4_	[19]
Rhodamine B	+	MWCNT/polyacrylonitrile	[139]
		TiO_2_/Cu-MOF	[91]
